# An Alternative Theory of Binocularity

**DOI:** 10.3389/fncom.2019.00071

**Published:** 2019-10-09

**Authors:** Cherlyn J. Ng, Dale Purves

**Affiliations:** ^1^Neuroscience and Behavioral Disorders Program, Duke-NUS Graduate Medical School Singapore, Singapore, Singapore; ^2^Flaum Eye Institute, University of Rochester, Rochester, NY, United States; ^3^Center for Visual Science, University of Rochester, Rochester, NY, United States; ^4^Duke Institute for Brain Sciences, Duke University, Durham, NC, United States

**Keywords:** vision, binocular, stereopsis, fusion, ocular dominance, correspondence problem, neural networks

## Abstract

The fact that seeing with two eyes is universal among vertebrates raises a problem that has long challenged vision scientists: how do animals with overlapping visual fields combine non-identical right and left eye images to achieve fusion and the perception of depth that follows? Most theories address this problem in terms of matching corresponding images on the right and left retinas. Here we suggest an alternative theory of binocular vision based on anatomical correspondence that circumvents the correspondence problem and provides a rationale for ocular dominance.

## Introduction

Many animals that are preyed upon sacrifice the advantages of fusion and stereopsis for the benefits of a wider field of view; in contrast, many predators sacrifice a more extensive view for the advantages of stereovision. For animals in the latter category, understanding vision therefore requires an explanation how information provided by the right and left eyes is brought together by the visual system. Particular challenges have been understanding (1) how information from the right and left eyes in animals with overlapping fields is fused; (2) how fusion generates stereopsis; and (3) why ocular dominance is characteristic in such animals. These puzzles have often been approached independently, primarily because no unifying scheme for this phenomenology has emerged. Here we outline a theory of binocularity that addresses these challenges based on anatomically corresponding points on the two retinas rather than corresponding image points.

### Models of Binocular Combination

That animals with overlapping visual fields take advantage of slight differences in the right and left eye images to generate an accurate sense of depth has been accepted since Charles Wheatstone's seminal experiments in the nineteenth century (Wheatstone, 1838, 1852). Fusion requires that inputs arising from the two eyes be combined by binocular neurons in the primary visual cortex. Although binocular cortical neurons have been thoroughly studied over the last 50 years (Hubel and Wiesel, 1962; Barlow et al., 1967; Nikara et al., 1968; Poggio and Fischer, 1977; Poggio et al., 1988; Poggio, 1995), how the visual system combines the right and left retinal inputs remains unclear.

**Figure 1:**
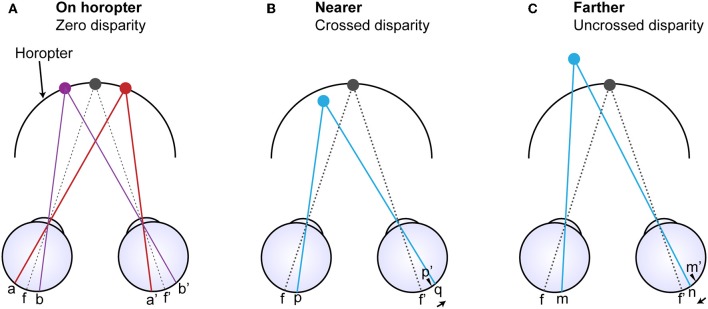
Diagram comparing corresponding anatomical points on the retinas and corresponding image points (Purves and Lotto, 2011). **(A)** The horopter is defined by all the points in visual space that fall on corresponding retinal loci (a and a' and b ad b') whether the points are considered from the perspective of images or retinal anatomy. Points f and f' are the foveal centers and thus the point of binocular fixation. **(B)** A light ray arising from a point nearer than the horopter (blue dot) projects onto point (p) in the left eye but onto point (q) in the right eye whose locus differs from the location of the anatomically corresponding point (p'). **(C)** Similarly, a light ray arising from a point more distant than the point of fixation falls on point (m) in the left eye but on point (n) in the right eye that also differs from the anatomically corresponding point (m'). Thus, image correspondence and anatomical correspondence are fundamentally different aspects of retinal disparity, and both could convey information about the depth of points in visual space relative to the horopter.

Theories that address stereopsis are generally based on the assumption that image points in one eye must be matched with corresponding image points in the other eye (Figure 1). A difficulty in this formulation, however, is explaining how left and right eye retinal neurons responding to the same physical point in space are linked, a challenge referred to as the “correspondence problem” [i.e., a mechanism that could match the anatomically unrelated points (p) and (q) and points (m) and (n) in Figures 1B,C]. As evidenced by physiology, some models seek to resolve this problem by limiting the relevant search space over multiple spatial scales (Marr and Poggio, 1979; Nishihara, 1984; Li and Atick, 1994a). Other models have been based on facilitation and inhibition (Dev, 1975; Nelson, 1975; Marr and Poggio, 1979; Mayhew and Frisby, 1980; Grossberg and Marshall, 1989). Inhibition is also featured in models that explain the related phenomenon of binocular rivalry (Lehky, 1988; Mueller, 1990; Dayan, 1998; Stollenwerk and Bode, 2003; Wilson, 2003; Freeman, 2005; Moreno-Bote et al., 2010). Said and Heeger (2013) have recently united these observations by incorporating elements of the stereoscopic model proposed by Li and Atick (1994b) to explain rivalry. Yet other models explain how the eyes could combine imbalanced but similar images; for example, by quadratic contrast summation (Legge, 1984) or interocular gain control that is in proportion to contrast energy (Ding and Sperling, 2006; Ding et al., 2013; Zhou et al., 2014).

Most salient, however, has been the so called spatio-temporal energy model proposed about 30 years ago (Ohzawa et al., 1990, 1997), which was adapted from an earlier model developed to address the “motion correspondence problem” (Adelson and Bergen, 1985). “Energy” refers to outputs of cells that are taken to compute a localized Fourier transform using two Cartesian coordinates and a temporal axis to account for motion. The model entails two stages. The first describes how monocular contrast is computed in simple cells related to one eye or the other; the second describes how matching monocular information is combined in complex binocular neurons (Ohzawa et al., 1990; Qian, 1997; Qian and Zhu, 1997). Since image differences in the two eyes vary with distance from the observer, binocular neurons could represent depth as the difference between left and right eye receptive field position and phase that give rise to the maximum binocular cross-correlation. Additional modifications have since been made to explain more recent observations (Read et al., 2002; Tanaka and Ohzawa, 2006; Haefner and Cumming, 2008; Tanabe and Cumming, 2008; Allenmark and Read, 2011), but there is still substantial support for cross-correlation being a major component of binocular processing.

Much of the evidence for cross correlation models has come from studies using random dot stimuli (Cisarik and Harwerth, 2008; Doi et al., 2011; Henriksen et al., 2016a,b; Goncalves and Welchman, 2017; Read and Cumming, 2019). Random dots are useful in that they offer no monocular cues to depth, yet enable control of the degree of correlation between stereo-images. Taken together with non-linear neuronal properties, the consensus has been that stereoscopic performance improves with greater inter-ocular correlation. Conversely, anti-correlated stimuli evoke small, oppositely tuned physiological responses (Cumming and Parker, 1997; Neri et al., 1999) but not psychophysical depth percepts (Cumming et al., 1998). More recently, these findings have been confirmed using noise stimuli (Reynaud and Hess, 2018) and natural images (Goncalves and Welchman, 2017).

### An Alternative Theory Based on Anatomical Correspondence

**Figure 2:**
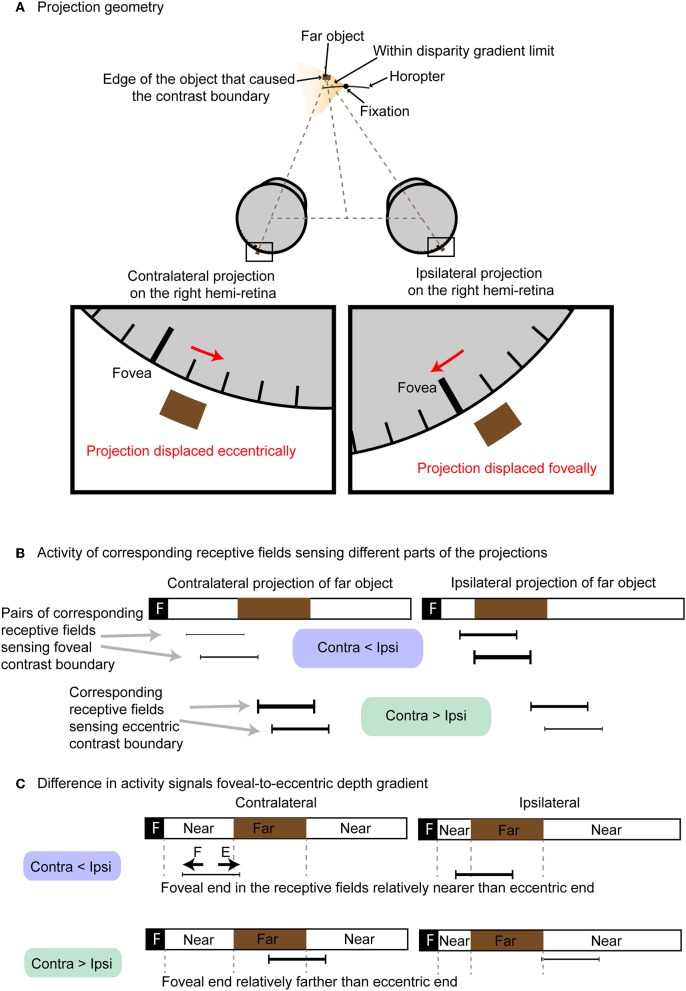
Different levels of neuronal activation based on anatomical correspondence. **(A)** Projection of an object farther than the horopter onto neurons at corresponding anatomical loci. The contralateral projection is displaced eccentrically, while the ipsilateral projection is displaced foveally. **(B)** The activity of neurons at anatomically corresponding receptive fields (black bars) differs. Except for projections of points in visual space that lie on the horopter, anatomically corresponding neurons in the two retinas will have different levels of activity (thickness of the black bars denote relative activity). **(C)** Difference in activity between contralateral and ipsilateral conveys whether the contrast boundary was formed by a near object on the foveal side of the receptive field (“F” denotes foveal half of the receptive field; “E” denotes eccentric half of the receptive field). Contralateral activity is less than ipsilateral activity when the eccentric side of the corresponding receptive field receives far stimuli. Conversely, ipsilateral activity is greater than contralateral when the eccentric side of the corresponding receptive field receives near stimuli.

Regardless of the type of stimuli used, contrast edges are essential for disparities to be perceived (i.e., there is no depth perception in a featureless field). The alternative theory we consider here is that binocular vision depends on differences in neuronal activity generated by contrast edges at anatomically corresponding retinal points (i.e., points a and a', b and b', p and p', and m and m' in Figure 1). The basis for this alternative scenario is that retinal disparity as a function of depth causes the distribution of light falling on corresponding anatomical points on the two retinas to differ routinely as shown in Figure 2.

Figure 2A shows how a far object within the disparity gradient limit in the left visual field projects onto anatomically corresponding points on the two retinas. Figure 2B shows the different activity of four pairs of neurons with corresponding receptive fields (black bars linked by gray dotted lines) as examples. These four pairs are illustrated because they represent neurons that detect the contrast edges to varying degrees. The relative activity of these pairs of anatomically corresponding neurons falls into two groups: (1) neurons in which the activity of the contralateral neuron is greater than the activity of ipsilateral neuron when the edge is formed by a near object at the foveal side of the receptive field and a far one at the eccentric side of the field; and (2) neurons in which the activity of ipsilateral neuron is greater than the activity of the contralateral neuron when stimulated by the opposite (edge formed by far object at the foveal side of the receptive field and near object at the eccentric end; Figure 2C). As a result, the relative activity at anatomically correspondence can convey information about depth at specific retinotopic points. Note that in this alternative theory the image correspondence problem does not arise. Planar image resolution can also be maintained without loss of spatial pooling across position or phase shifts.

## Methods

**Figure 3:**
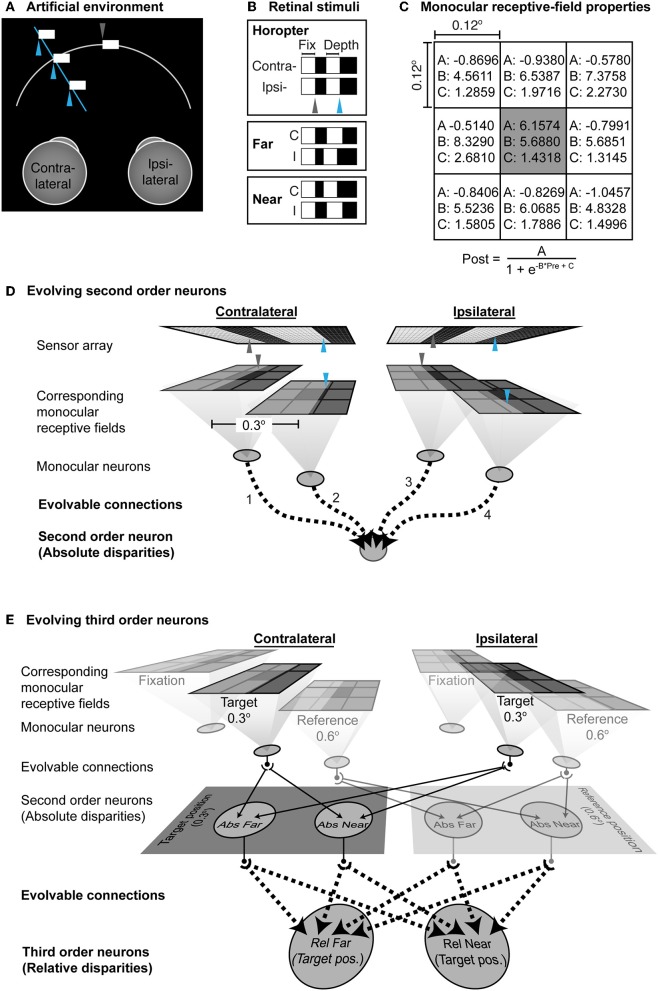
Artificial neural network trained on the basis of anatomical rather than image correspondence. **(A)** The environment. The fixation point is a locus on a contrast boundary in the midline (gray arrowhead) at a constant distance; another contrast boundary (blue arrowhead) varied in depth along a particular line of sight to the left of the fixation point. **(B)** The stimuli falling on the two retinas in this arrangement. **(C)** Parameters of the monocular receptive field. **(A–C)** are pre-evolved sub-region specific parameters (Morgenstern et al., 2014). The input to the sigmoid in each subregion is the average luminance within a grid. **(D)** The network used for evolving the functionality of second order neurons. The projection of the contrast boundary at the point of fixation is indicated by the gray arrowheads; the blue arrowheads indicate the projection of the contrast boundary in depth. The corresponding monocular neurons have receptive fields at identical “anatomical” positions on the left and right sensor arrays. The outputs of the monocular neurons provided the inputs to a second order neuron to could evolve to either report far- or near-absolute disparities. Evolvable connections are shown by dotted lines. **(E)** Network used to evolve third order neurons. The evolved network that could successfully report absolute disparities was cloned at the reference position. Dotted lines indicate evolvable connections from the second order to the third order neurons.

To examine the circuitry that would be required to implement this theory of binocularity, we created a simple, simulated environment where artificial neural networks were trained on the basis of information arising from anatomical rather than image correspondence (Figure 3).

### Environment

The simulated environment comprised two uniformly illuminated fronto-parallel contrast boundaries (400 cd·m^−2^ against a 1 cd·m^−2^ background). One contrast boundary included the point of fixation along the midline; the other was 0.3° to the left and varied in depth along the line of sight of the fused stimulus (see Figures 3A,B). The surfaces were projected through pinholes onto a pair of 2-dimensional sensor arrays as shown in Figure 3B. Each array was 200 × 40 pixels, spanning 2° horizontally and 0.4° vertically. The vertical boundary at the midline including the fixation point spanned 0.3 × 0.4° whereas the size of the other surface varied with its depth. Thus, stimuli generated by the environment gave rise to contrast boundaries that fell at different locations on the receptor array.

### Network

The sensor arrays comprised pixels 0.01 × 0.01° and represented photoreceptors that received images projected from the environment. The sensors activated two pairs of anatomically corresponding monocular neurons whose center-surround receptive fields were 0.3° apart. The receptive field properties of these monocular neurons were pre-set according to Morgenstern et al. (2014) (Figure 3C). One pair of corresponding neurons received luminance signals from the temporal edge at fixation (gray arrowheads in Figure 3D), while the other pair received luminance signals from the contrast boundary of the surface that varied in depth (blue arrowheads). Since the stimuli presented a positive contrast boundary with respect to the background, the monocular neurons in the network were modeled as ON-center surrounded by eight OFF-center sub-regions. The entire receptive field spanned 36 × 36 pixels on the sensor (i.e., the “photoreceptors”) equivalent to 0.36° visual angle. The receptive field centers were 0.12° in diameter (12 × 12 pixels) surrounded by eight 0.12° inhibitory sub-regions. Hence each receptive field can be thought as a quantized Gabor function. Each sub-region transformed the average luminance that fell within it using the different sigmoidal connection parameters specified in Figure 3C and Morgenstern et al. (2014). A final sigmoid with parameters (A: 0.9771, B: 5.7152, C: 1.7300) compiled the sum of these individual transformations as the output of the network's monocular neurons. Other than these pre-set monocular parameters, all other downstream connections were evolvable.

The outputs of the network's monocular neurons provided inputs to second order binocular neurons via evolvable synaptic connections (Figure 3D). Networks were initialized with weak but fully connected feedforward connections. Depending on functionality, any of these connections could be lost during evolution, or become excitatory or inhibitory with different strengths on the basis of the equation

(1) $$\text{Post}=\frac{\pm \text{A}}{\left(1+{\text{e}}^{-\text{B}\times \text{Pre}+\text{C}}\right)}$$

Thus parameters A, B and C were freely evolving parameters as in (Ng et al., 2013). “Pre” is the input to the sigmoid and “Post” is the result of each sigmoidal transformation. The summed effect of all these connections represented the second order neuron's activity as

(2) $$\text{Binoc}=\;\sum_{\text{c}=1}^{4}\text{Post}$$

where “Binoc” is the activity that signifies the depth of the contrast boundary; “c” represents the connections from each of the four monocular neurons in the network, and Post the summed effect. At the end of evolution, the second order binocular neurons reported whether the contrast boundaries were nearer or farther than the horopter and by how much (i.e., absolute disparity).

The second order binocular neurons were cloned at a further 0.3° eccentric to the original position (i.e., 0.6° in total from the fixation point). The position of these clones was called the “reference position” and the originally evolved position the “target position.” An additional layer of third order binocular neurons was also added (Figure 3E). All second order neurons at both target and reference positions were fully connected to the third order neurons with evolvable sigmoids as described above. These third order binocular neurons evolved in turn to report the relative disparity of the object at the target position (0.3° from the fixation point) with respect to the object at the reference position (0.6° from the fixation point), based on the absolute disparity reported by the second order neurons.

### Evolution

Binocular stimuli were presented to 20 populations of 500 individually evolving networks. Two hundred stimuli were presented to each network in an evolving population during the network's “lifetime.”

The success of the second order neurons was measured by how well its output approximated the absolute disparity of the object surface relative to the fixation point using the formula

(3) $$\begin{array}{l} {\text{Success}}_{\text{k}}=\left(\sum_{\text{n}}\left|{\text{Response}}_{\text{k}}{-\text{Disparity}}_{\text{k}}\right|\right)\\ \;-\left(\left|{\text{Response}}_{\text{i}}{-\text{Disparity}}_{\text{i}}\right|\right) \end{array}$$

where “k” represents the kth individual in the population and “i” the i-th trial presented to each network. “Response” denotes the network's response on a given trial; “Disparity” is the absolute disparity in degrees of visual angle from the object boundary to the fixation point, and “Pop” the population size. Networks that discriminated near surfaces were evolved separately from those that discriminated far objects.

Second order neurons were evolved separately and prior to the third order neurons, their evolved outputs serving as inputs to third order neurons. The success of the evolving third order neurons was evaluated in the same way except that “Disparity” was now the relative disparity of the contrast boundary at the target position with respect to the boundary at reference position.

At the end of each generation all the networks in a population were ranked in order of their success determined by Equation (3). Each network was assigned a sector on a roulette wheel with a size proportional to its success score. The wheel was then spun 500 times to choose the networks that populated the next generation. Accordingly the more successful networks were selected often and the less successful networks only occasionally. The connection parameters of each individual in the new population had an 80% chance of being randomly exchanged with those of another network to introduce novelty and diversity (Ng et al., 2013; Morgenstern et al., 2014). Performance was then calculated again, and the process repeated for 2,500 generations. All simulations were performed using the Genetic Algorithm in the Matlab Global Optimization Toolbox.

## Results

### Implications for Visual Circuitry

**Figure 4:**
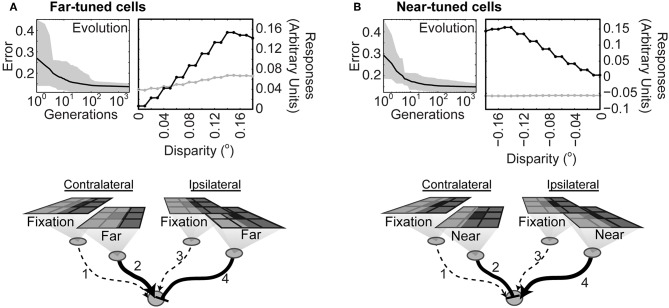
Diagram of connections after the networks had learned to correctly report differences in stimulus strength at anatomically corresponding loci on the input arrays. **(A)** Top left: Evolution of networks trained to respond to contrast boundaries farther than the horopter. Black line is the average error across all the populations tested; the gray overlay shows the absolute deviation from the mean. (Top right): Responses before (gray) and after (black) evolution. Bottom: Average connectivity of the evolved networks. The strength of the connections is indicated by the thickness of the lines; solid lines with arrowheads represent excitatory connections and dotted lines connections with little or no effect. Inhibitory connections are denoted by the end-stopped lines. **(B)** Evolution and average evolved connectivity of networks trained to respond to contrast boundaries nearer than the horopter. Evolved parameters are given in Supplementary Table 1.

After 2,500 generations the output of the second order neurons had learned to specify whether a stimulus boundary was nearer or farther than the horopter and by how much (Figure 4). Networks that identified contrast boundaries farther than the horopter evolved a strong excitatory connection from the contralateral monocular neuron, but not from the ipsilateral neuron (Figure 4A). Conversely, networks that discriminated stimuli arising from boundaries nearer the horopter evolved a strong excitatory connection from the ipsilateral neuron, but not from the contralateral neuron (Figure 4B). In both instances the other monocular input to the second order neuron was inhibitory. In a control simulation to test the importance of retinotopy, networks were free to evolve depth responses to stimuli placed anywhere within the visual field. Such networks failed to evolve (Supplementary Figure 1), showing that responses to disparity had to be tied to specific locations in the visual field sampled by the relevant anatomically corresponding neurons.

**Figure 5:**
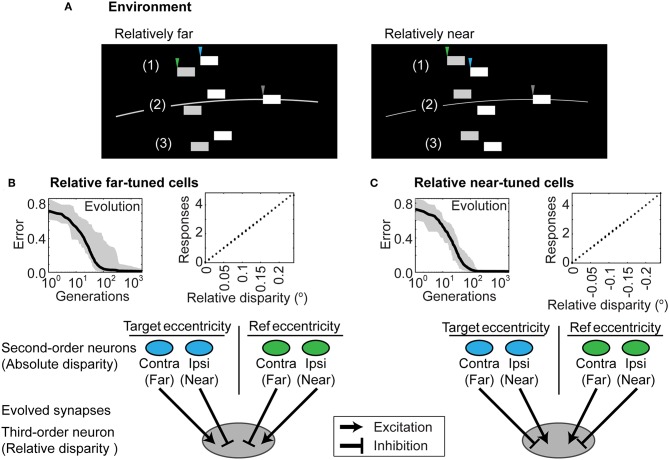
Distinguishing relative depth. **(A)** Stimuli that entail relative distances in depth for two contrast boundaries that (1) are both farther than the horopter; (2) straddle the horopter; or (3) are both nearer than the horopter. All three instances make up the stimulus pool in training relative disparity discrimination. In the examples shown, one boundary is relatively nearer than the other (left panel) or relatively farther away (right panel). As in Figure 3, gray arrowheads indicate the contrast boundary at fixation; the blue arrowheads indicate the original target contrast boundary in Figure 3D while the green arrowheads indicate an additional reference contrast boundary to which the depth of the original stimulus is compared (see Figure 3E). **(B)** Networks that evolved to report relative disparities that were farther than the object at reference position. **(C)** Networks that evolved to report relative disparities that were nearer than the object at reference position. Each connection was comparable in strength, as indicated by the similar thickness of the arrows. The evolved parameters are shown in Supplementary Table 2.

**Figure 6:**
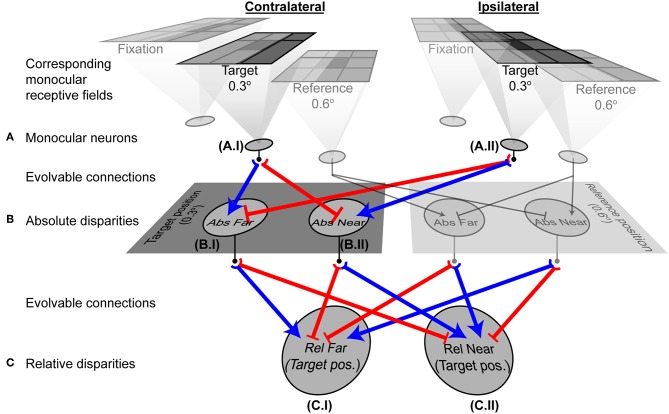
A fully evolved network. Shaded neurons indicate adjacent units at the reference position that were included in the architecture, but whose responses were not considered in the analyses because their receptive fields were not at the relevant target position. Neurons at the target position (0.3° from the fixation) are outlined in bold and their evolved connections shown in color. Blue arrows are excitatory connections; red end-stopped lines indicate inhibitory connections. **(A)** Three corresponding pairs of monocular neurons receiving visual information. The outputs of neurons A.I and A.II contributed to the evolved responses of the absolute disparity neurons B.I and B.II. **(B)** Second order absolute disparity neurons at the target (B.I and B.II) and reference positions. Outputs of these neurons contributed to the relative disparity neurons C.I and C.II. **(C)** Third order relative disparity neurons at the target position. These neurons reported how far or near the target was, with respect to the reference object. Graphs in Figure 7 and Supplementary Figure 2 show the detailed responses from the neurons here.

The connectivity of the two-layer network after training could not, however, distinguish the relative disparity of two contrast boundaries both of which were closer or farther than the horopter, or that straddled the horopter (Figure 5A). To address this further challenge, an additional (third) layer in the network was tasked with making these further distinctions (see Methods and Figure 3D). Given this addition, networks using the responses generated by the two-layer network in Figure 3 successfully reported relative depth (Figures 5B,C). The complete network after training is shown in Figure 6. The relevant monocular neurons at the target position are shown in Panel A, labeled as A.I and A.II. These fed into the second-order absolute disparity units the evolved at the target position (Panel B; neurons labeled as B.I and B.II), which along with those at the reference position, were inputs to the third order relative-disparity neurons (Panel C; neurons C.I and C.II). Throughout the network, the evolved mechanism underlying successful evolution was antagonistic connections (blue and red lines). Thus, the third order relative far-tuned neurons were excited by the contralateral neurons at the target position, as well as the by the ipsilateral neuron at the reference position. These relative far neurons were also inhibited by the ipsilateral and contralateral neurons at the respective positions (Figure 6C, left). In contrast, relative near-tuned neurons were excited by the ipsilateral neuron of the target and the contralateral neuron of the reference, but inhibited by the target's contralateral and the other ipsilateral neurons (Figure 6C, right). These excitatory and inhibitory connections had almost equal effects on the relative disparity neurons (Supplementary Table 2), and signals in the third order neurons were brought about by differences in the activity of the second order neurons. This arrangement thus reported not only relative disparity magnitudes, but also the comparative retinotopic positions.

### Ocular Dominance

Ocular dominance refers to the fact that most binocular neurons in the primary visual cortex of carnivores and primates are more strongly driven by one eye or the other. Despite its discovery more than 50 years ago, a rationale for ocular dominance has never been specified. Although dominance has been widely used in experimental animals as an index of cortical connectivity during development (Hubel and Wiesel, 1962, 1965, 1970) or to better understand and treat strabismus in clinical ophthalmology (Horton, 1992), its purpose in vision, if any, has remained unclear. The association of ocular dominance and binocular vision is nonetheless obvious, leading some investigators to speculate that it must play some role in binocular vision (Hubel and Wiesel, 1962; Gardner and Raiten, 1986; LeVay and Voigt, 1988). Nevertheless, the consensus more recently has been that ocular dominance and its anatomical expression as cortical columns or stripes in carnivores and primates have no particular function (Purves et al., 1992; Read and Cumming, 2004; Horton and Adams, 2005).

In the present theory ocular dominance arises naturally from binocularity based on anatomical correspondence. Thus, after training, the second order binocular neurons were more strongly driven by one eye or the other, as shown in Figure 4. Being excited by either contralateral or ipsilateral input and inhibited by the other input, the evolved units resembled early stage V1 neurons. Moreover, these second order units would have been organized into groups if tiled across the whole visual field. The anatomical model also accords with physiological observation that disparity tuned cells tend to be dominated by one eye or the other (Poggio and Fischer, 1977; Ferster, 1981; LeVay and Voigt, 1988) whereas non-disparity tuned cells tend to be equally driven by both eyes (Gardner and Raiten, 1986). Much as we observed in the evolved networks, far disparities in experimental animals are associated with contralateral dominance and near disparities with ipsilateral dominance (LeVay and Voigt, 1988).

In contrast to the second order binocular neurons, the third order binocular neurons showed little or no correlation between depth tuning and ocular dominance (*r* = 2.3 × 10^−3^, *p* > 0.05 for far-tuned neurons; *r* = 4.2 × 10^−3^, *p* > 0.05 for the near-tuned neurons). In short, ocular dominance only arises as a consequence of low-level absolute disparity computations but not computations of relative disparity. This finding is also in line with balanced ocularity apparent higher in the visual pathway of experimental animals.

### Further Comparisons With Physiology

Many studies have related the disparity tuning curves of biological neurons to receptive field sizes (Marr and Poggio, 1979; Tsao et al., 2003; Nienborg et al., 2004). There is also psychophysical evidence from the size-disparity correlation (Richards and Kaye, 1974; Schor and Wood, 1983; Smallman and McLeod, 1994). Marr and Poggio in particular have pointed out a simple mechanism that avoids false matches if the disparity range is within half of the receptive field width. In keeping with these results, we observed that the second order units evolved to follow this rule (the upper disparity limit being ~±0.14° compared to 0.36° receptive field size), also in agreement with the π phase limit of macque V1 neurons (Prince et al., 2002). Since third order neurons combined both near and far disparities, the upper disparity limit was twice that of the second order neurons (Figures 5B,C). The receptive field size of third order neurons was also bigger (0.66°) because of spatial pooling. Hence, the upper disparity limit was still approximately half of the receptive field size.

The monocular (Figures 6A.I,II), as well as the evolved second order units (Figures 6B.I,II) also display phase dependency characteristic of simple cells. Moreover, phase dependency was not observed in the third order units (Figures 6C.I,II), analogous to visual neurons further on in the binocular processing hierarchy (Supplementary Figure 2).

**Figure 7:**
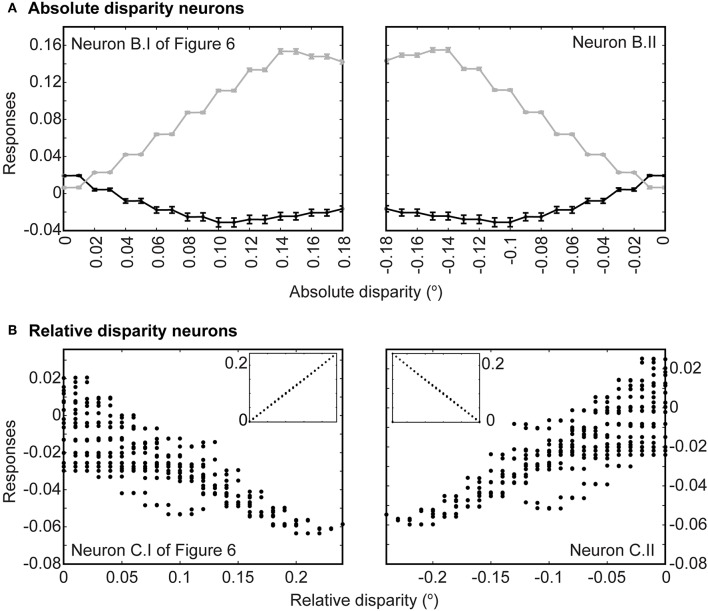
Responses to anti-correlated patterns. **(A)** Responses at the level of absolute disparity neurons in Figures 6B.I,II. Gray traces are responses to conventional stereograms as a function of disparity; black traces are responses to anti-correlated patterns. Each point indicates the average response across all 20 populations tested; error bars are standard deviations. **(B)** Responses to anti-correlated stereograms at the level of relative disparity neurons (Figures 6C.I,II). Each point represents an average response from 20 populations to a particular absolute disparity combination. Each relative disparity could have been obtained from many combinations of absolute disparities; hence there are multiple points for each position on the abscissa. Insets show responses to regular stereograms.

Finally, we presented anti-correlated input patterns to see if the networks showed the disparity tuning reversals evident in binocular neurons in the primary visual cortex (Cumming and Parker, 1997; Neri et al., 1999). In agreement with physiological observations, our networks showed small, reversed tuning when presented with anti-correlated luminance profiles. These reversals were apparent at both the level of the absolute disparity neurons and the relative disparity neurons. Figure 7A shows the tuning function of the evolved absolute far (left panel) and absolute near (right panel) neurons. In both cases, responses to conventional stereograms are plotted in gray and show much greater dynamic ranges (between 0 and 0.15) than responses to anti-correlated stereograms (0.02 to −0.03). Most responses to anti-correlated stereograms were also negative and hence inhibitory for the same disparity range, rising only slightly above the null point toward zero disparity and the opposite depth polarities (i.e., near disparities for far-tuned neurons and vice versa). These reversals were also evident for the evolved third order neurons (Figure 7B). Here, multiple responses could have resulted from a single relative disparity (many ordinate points for one abscissa reading in Figure 7B; see figure legend). Like the second order absolute disparity neurons, responses to anti-correlated stereograms were also small (0.02 to −0.06) compared to conventional stereograms (0–0.2; insets in Figure 7B), largely inhibitory, and only rose to become slightly excitatory when disparities tended toward the opposite polarities.

## Discussion

### Limitations

The theory we outline here has obvious limitations that would need to be examined before it could compete with or even replace existing theories and models. Some of these limitations are:

1. The minimal stimuli we used. The theory would have to be tested with more complex (and eventually natural) images.
2. Although the correspondence problem is resolved by the present anatomical theory the problem of “false matches” would also need to be explored.
3. The model we outline would eventually have to be tested using random dots stereograms.

The anatomically grounded mechanism we propose is not necessarily in conflict with cross-correlational theories. Disparity signals from cross-correlation could combine with or be supplemented by information from anatomical correspondence.

### Relevance to Visual Perception

Binocular visual phenomena (e.g., summation, rivalry, and stereoscopic depth) are perceptions. An important question, therefore, is how the present theory aligns with understanding of the perception of other visual qualities, such as luminance, color, geometry and motion. In the case of these basic qualities recent work has indicated that perceptions arise from accumulated experience (Purves and Lotto, 2011; Ng et al., 2013; Purves et al., 2014; Purves, 2019). In each of these categories, experience gives rise to empirical “scales” that determine what is actually seen, suggesting that the same framework may underlie binocular phenomenology. For example, the explanation of stereopsis in terms of anatomical correspondence may be empirically generated associations that link differences in the levels of activity at the same retinotopic loci with differences in perceived depth relative to the horopter. Similar to other visual percepts, fusion and perceived depth may follow from experience with different levels of activity at corresponding retinal points.

## Conclusion

With the exception of points on the horopter, the frontal eyes of carnivores and primates require that loci in visual space project to different anatomically corresponding points on the right and left hemi-retinas. In consequence, unequal monocular activity arises in neurons at retinotopically corresponding loci whenever the generative physical points are nearer or farther than the horopter. When conveyed to binocular neurons, this differential activation can specify the magnitude and direction of both absolute and relative depth. This alternative theory is consistent with observations in experimental animals, circumvents the image correspondence problem and may explain why ocular dominance is only apparent in animals with stereo vision.

## Data Availability Statement

Matlab programs used to generate the artificial environment and stimuli, as well as evolve the absolute and relative disparity neurons and analyze the results are found in doi: 10.5281/zenodo.3401688.

## Author Contributions

CN and DP conceived the hypothesis and wrote the paper. CN designed the ANN model.

### Conflict of Interest

The authors declare that the research was conducted in the absence of any commercial or financial relationships that could be construed as a potential conflict of interest.
