# Supplementary material for: An Alternative Theory of Binocularity
Source: Front Comput Neurosci. 2019 Oct 9;13:71. doi: 10.3389/fncom.2019.00071 (PMC6794442; doi:10.3389/fncom.2019.00071)
Supplement: Supplementary file 1 [file Table_1.PDF]

Supplementary Material

1 Supplementary Tables

Supplementary Table 1. Average synaptic parameters in the evolved absolute disparity networks to (A) far and (B) near disparities. After 2500 evolutionary generations, the three evolvable parameters at each connection were recorded for the best performing networks. The mean and SEM parameters across all populations are shown. Connections are labeled as in Figure 3 and 4.

(A) Absolute-far

**Parameter**	**Connection**	
	**2**	**4**
A	1.1958 ± 0.1014	-0.7872 ± 0.1182
B	1.1092 ± 0.1836	0.0260 ± 0.0765
C	1.2276 ± 0.4611	0.3077 ± 0.5908

(B) Absolute-near

**Parameter**	**Connection**	
	**2**	**4**
A	-0.8051 ± 0.1425	1.2108 ± 0.1165
B	0.0149 ± 0.0371	1.1438 ± 0.1703
C	0.3966 ± 0.7219	1.3304 ± 0.4823

Supplementary Table 2. Average synaptic parameters in the successfully evolved relative disparity networks to (A) relative-far and (B) relative-near disparities. After 2500 evolutionary generations, the three evolvable parameters at each connection were recorded for the best performing networks. The mean parameters and SEM across all populations are shown. Connections are labeled as in Figure 5.

(A) Relative-far

Parameter	Connection			
	Target-Contra	Target-Ipsi	Other-Contra	Other-Ipsi
A	1.6707 ± 0.7488	-0.9937 ± 0.1960	-0.9166 ± 0.5332	1.3717 ± 0.4836
B	3.4959 ± 0.6754	4.9397 ± 1.9644	4.2618 ± 1.4912	3.8706 ± 1.7863
C	1.0330 ± 0.8003	0.2803 ± 0.6589	0.3982 ± 0.6049	0.7094 ± 0.6377

(B) Relative-near

Parameter	Connection			
	Target-Contra	Target-Ipsi	Other-Contra	Other-Ipsi
A	-1.1714 ± 0.3448	1.8087 ± 0.7681	1.6719 ± 0.5670	-1.0140 ± 0.5178
B	4.4093 ± 1.5122	3.0352 ± 0.7292	3.2267 ± 0.7113	3.7939 ± 1.1969
C	0.3703 ± 0.7592	0.9226 ± 0.6784	0.9334 ± 0.6316	0.3627 ± 0.6327
