# Supplementary material for: An Alternative Theory of Binocularity
Source: Front Comput Neurosci. 2019 Oct 9;13:71. doi: 10.3389/fncom.2019.00071 (PMC6794442; doi:10.3389/fncom.2019.00071)
Supplement: Supplementary file 2 [file Image_1.PDF]

Supplementary Material

1 Supplementary Figures

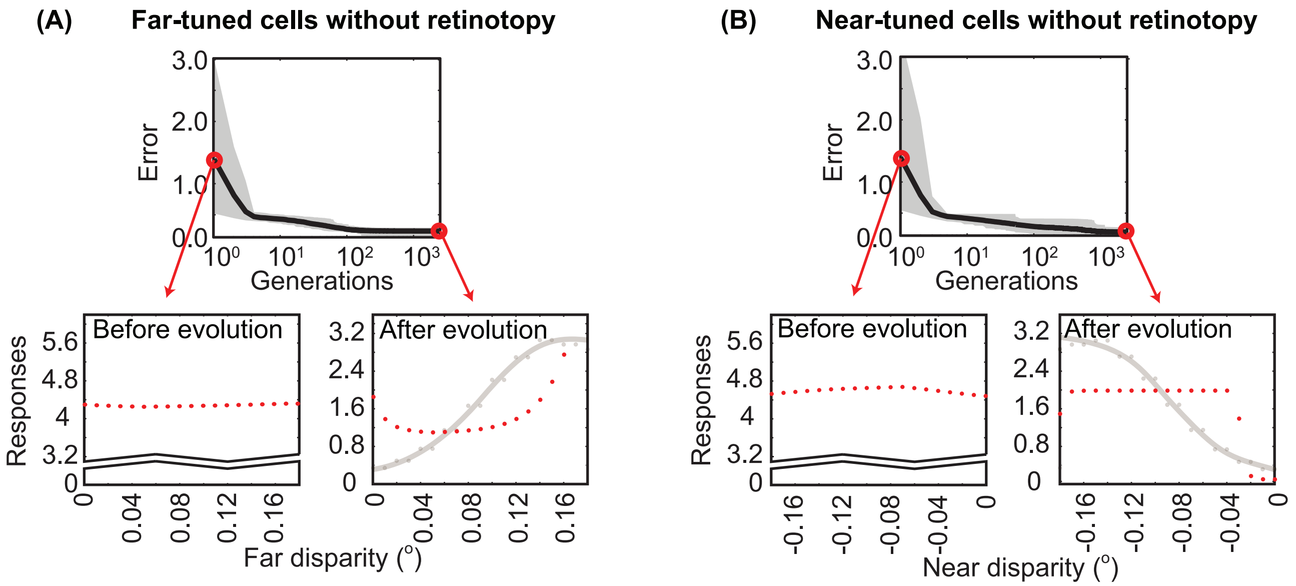

Supplementary Figure 1. The importance of retinotopy. (A) Average evolutionary profile of binocular neurons that received monocular inputs from the contrast boundaries of far objects placed at any eccentricity within 2° of the right visual hemi-field. Graphs below show the responses before and after 2,500 generations of evolution (red dots). Gray line indicates the average responses of networks that were retinotopically tuned to receive signals only from stimuli at 0.3° eccentricity (see Figure 3). (B) Average evolutionary profile and responses of binocular neurons that received monocular inputs from the contrast boundaries of near objects placed at any eccentricity within 2° of the right hemi-field. Thus networks not retinotopically tuned failed to evolve depth discrimination, even though depth signals from unequal binocular stimulation were present.

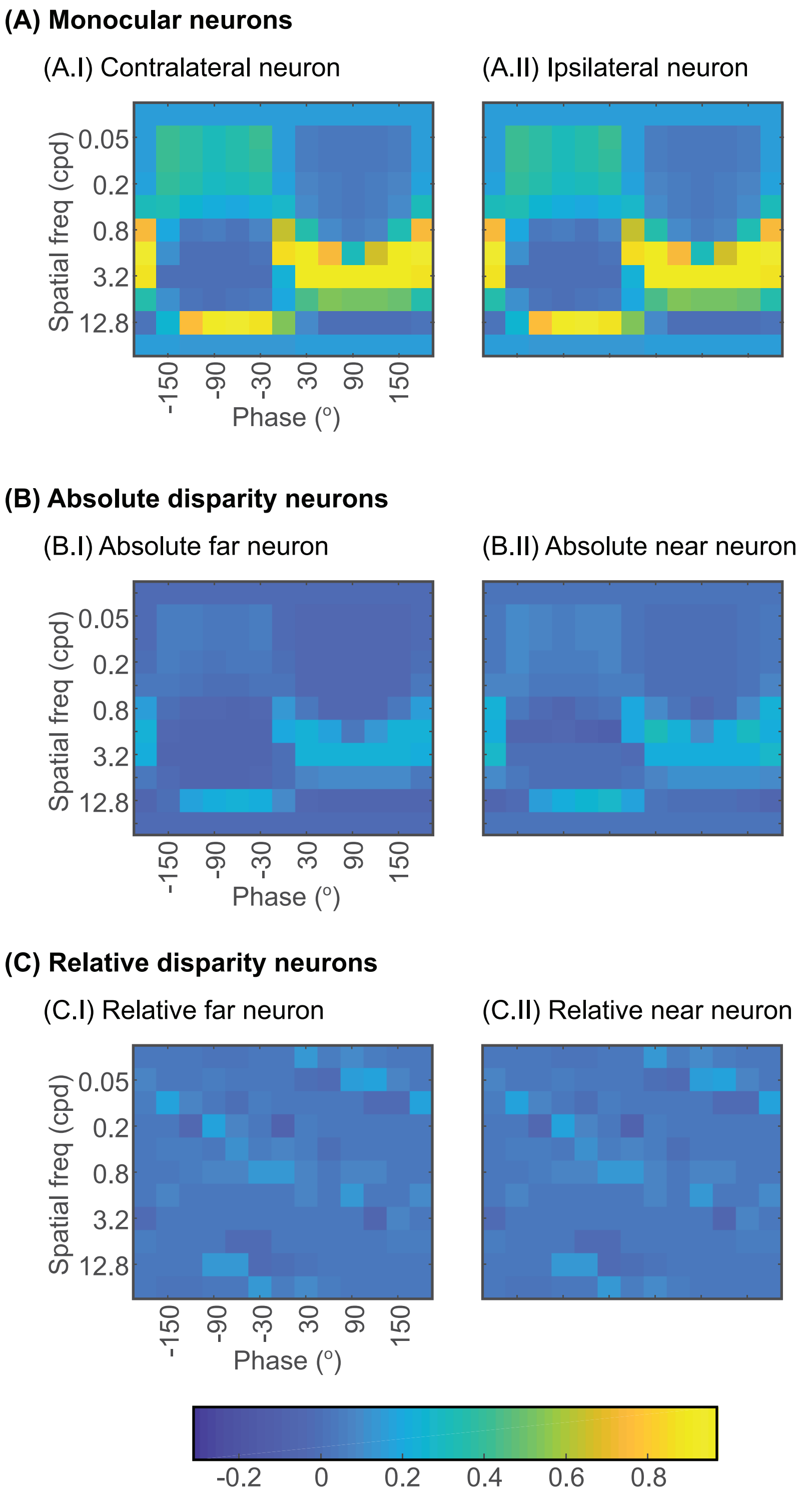

Supplementary Figure 2. Responses to full field vertical sine wave gratings. Phase was expressed relative to the position of the receptive fields of the target neurons; i.e., the 0° phase angle of a sine

wave grating is at 0.3° eccentric from the fixation point. Unlike relative disparity neurons, monocular and absolute disparity neurons show phase dependent responses. Labels correspond to neurons shown in Figure 6.
